# Neurological and Neuroimaging Features of *CYB5R3*-Related Recessive Hereditary Methemoglobinemia Type II

**DOI:** 10.3390/brainsci12020182

**Published:** 2022-01-29

**Authors:** Francesco Nicita, Letizia Sabatini, Viola Alesi, Giulia Lucignani, Ester Sallicandro, Antonella Sferra, Enrico Bertini, Ginevra Zanni, Giuseppe Palumbo

**Affiliations:** 1 Unit of Neuromuscular and Neurodegenerative Disorders, Genetics and Rare Diseases Research Division, Department of Neurosciences, Bambino Gesù Children’s Hospital, IRCCS, 00146 Rome, Italy; antonella.sferra@opbg.net (A.S.); enricosilvio.bertini@opbg.net (E.B.); ginevra.zanni@opbg.net (G.Z.); 2 Department of Pediatric Hemato-Oncology and Cell and Gene Therapy, Bambino Gesù Children’s Hospital, IRCCS, 00146 Rome, Italy; letizia.sabatini@opbg.net (L.S.); giuseppe.palumbo@opbg.net (G.P.); 3 Department of Pediatrics, University of Rome Tor Vergata, 00146 Rome, Italy; 4 Laboratory of Medical Genetics, Translational Cytogenomics Research Unit, Bambino Gesù Children 5 Hospital and Research Institute, IRCCS, 00146 Rome, Italy; viola.alesi@opbg.net (V.A.); ester.sallicandro@opbg.net (E.S.); 5 Unit of Neuroradiology, Department of Radiology, Bambino Gesù Children’s Hospital, IRCCS, 00146 Rome, Italy; giulia.lucignani@opbg.net

**Keywords:** microcephaly, ponto-cerebellar hypoplasia, cyanosis, brain atrophy, NADH-diaphorase, DIA-1, generalized methemoglobinemia, methemoglobin reductase

## Abstract

Recessive hereditary methemoglobinemia (RHM) due to NADH-cytochrome b5 reductase deficiency is a rare disease caused by pathogenic variants in *CYB5R3*. Unlike type I, in RHM type II (RHM2), the enzymatic defect affects erythrocytes and all body tissues, thus resulting in cyanosis and neurological impairment. Although the first description of RHM2 dates back to the mid-1950s, detailed clinical and neuroimaging information are available for only a few patients. Here, we describe a new patient with RHM2 that harbors an unreported homozygous 31 Kb deletion involving part of *CYB5R3*, and showing a peculiar neuroimaging pattern resembling a ponto-cerebellar hypoplasia-like condition. A careful review of the available literature was performed with the aim of better delineating neurological and neuroimaging as well as the genotypic spectra of this extremely rare disease.

## 1. Introduction

Recessive hereditary methemoglobinemia (RHM) due to NADH-cytochrome b5 reductase (NADH-CYB5R3) (MIM #250800) deficiency is a rare disease and the most frequent cause of congenital methemoglobinemia. It is caused by mutations in *CYB5R3* (MIM #613213), located on chromosome 22q13, for which its alternative splicing generates two isoforms [1]. The soluble erythrocyte isoform of CYB5R3 is involved in the reduction of methemoglobinemia (MetHb) to hemoglobin, thus restoring the oxygen-binding capacity of hemoglobin. The membrane-bound isoform is anchored to the mitochondrial outer membrane, endoplasmic reticulum and plasma membrane of somatic cells and is involved in several metabolic processes including fatty acid desaturation and elongation, cholesterol biosynthesis and cytochrome P450-dependent drug metabolism [2]. Studies on transcriptional and translational mechanisms of *CYB5R3* have clarified that the organization of the 5′ gene region is not conserved between humans and rodents and that insertion of Alu elements into the first exon of the human gene may have contributed to its dynamic evolution [3]. There are two types of NADH-CYB5R3 deficiency, depending on which isoform is involved. In type I (RHM1), the defect is restricted to the erythrocyte soluble form and causes a well-tolerated cyanosis usually with a mild clinical presentation and a normal life expectancy. In type II (RHM2), the deficiency affects both isoforms and impacts red cells and all body tissues, thus resulting in a cyanosis associated with neurological manifestations [1]. Beyond the previously established concept, according to which neurologic involvement in RHM2 is linked to abnormal lipid metabolism with subsequent neuronal demyelination, recently discovered functions of CYB5R3 in mammalians indicate that other mechanisms, such as synapse depression, vascular nitric oxide insensivity, mitochondrial homeostasis and NAD^+^ depletion, may also play a role [2]. Although the first description of RHM2 dates back to the mid-1950s, only a few patients have detailed clinical and neuroimaging information available. Here, we describe a new patient with RHM2, harboring an unreported homozygous 31 Kb deletion involving part of *CYB5R3*, and showing a peculiar neuroimaging pattern resembling a ponto-cerebellar hypoplasia (PCH)-like condition. A careful review of the available literature was performed, aimed at better delineating the neurological and neuroimaging phenotypes as well as the genotypic spectra of this rare disease.

## 2. Materials and Methods

### 2.1. Case Description

**Figure 1:**
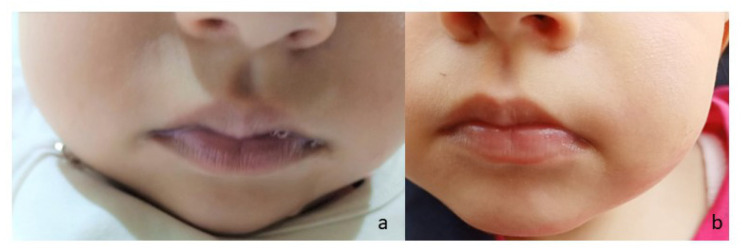
Pictures of patient before (**a**) and after (**b**) treatment with ascorbic acid showing complete resolution of the cyanosis of the lips (performed at 15 months and 20 months of age, respectively).

This 2-year-old girl is the second daughter of healthy and apparently non-consanguineous parents. There was no history of neurological or other inherited diseases in either family. She was born at term by cesarean delivery after an uneventful pregnancy. Apgar score was 9 at 5 min. Birth weight was 3560 gr (50–75%), birth length was 49.5 cm (25–50%) and head circumference was 35 cm (50–75%). No problems appeared during the neonatal period and first months of life. At around 7 months of age, cyanosis of the upper and lower extremities and lips (Figure 1a) was observed. Psychomotor delay was also evident since the patient did not control her head or produce vocal sounds. Alternating squints and abnormal head posturing were observed at 1 year of age.

**Figure 2:**
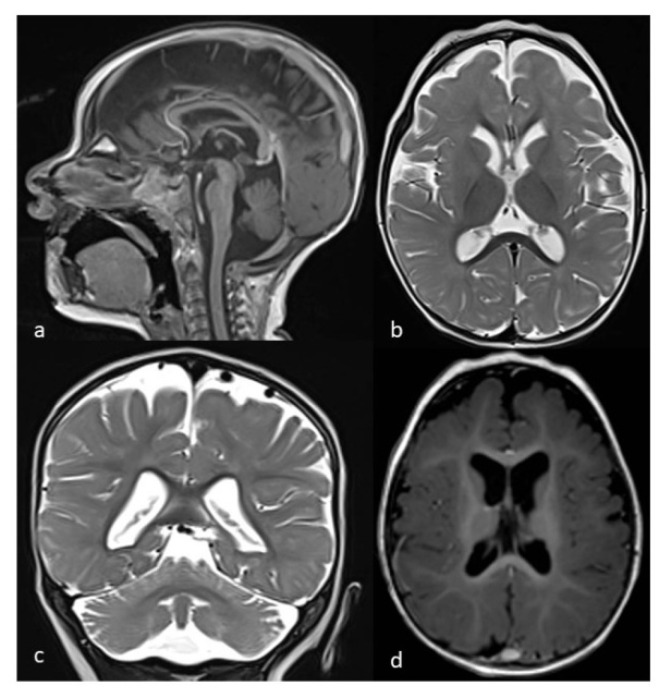
Brain MRI (T1-weighted in (**a**,**d**); T2-weighted in (**b**,**c**)) of the patient performed at age 14 months showing thin corpus callosum and hypoplasia of pons (**a**) and cerebellar vermis (**a**,**c**), simplified gyral pattern with reduction in frontal gyri and enlargement of sub-arachnoids spaces (**b**,**d**), mild reduction in the total amount of white matter (**b**,**d**) and enlargement of cerebellar interfolial spaces (**c**).

When she came to our attention at age 14 months, a neurological examination revealed the following: alternating squint; tetraparesis with appendicular spasticity and axial hypotonia; psychomotor delay with poor social eye contact; lack of vocalization; and incomplete head control. Evaluation according to the Griffiths Developmental Scale revealed a mental age of 6 months. Brain magnetic resonance imaging (MRI) revealed a complex malformative condition partially resembling PCH (Figure 2a–d). After sedation in the course of performing brain magnetic resonance imaging, the patient manifested desaturation at 82–83%, which scarcely increased after oxygen supplementation. Blood gas analysis indicated a MetHb of 20%. Methylene blue therapy was then administered at a dosage of 1 mg/kg with improvement of SpO2 (99%) and MetHb (3%) values. Chest X-ray and heart ultrasound were normal. Since a recent history of repetitive dietary nitrate consumption was present, an acquired cause of methemoglobinemia was initially considered, although genetic analyses were also conducted due to neurological involvement. Signed written informed consent for genetic analysis and for study participation was obtained from the patient’s parents.

### 2.2. Genetic Analysis

#### 2.2.1. SNP Array

DNA was extracted from the peripheral blood by means of a QIA symphony automatic extractor (QIAGEN, Hilden, Germany; www.qiagen.com, accessed on 15 January 2022). CMA (Chromosomal Microarray Analysis) was performed using Infinium CytoSNP-850 K BeadChip (SNP-array, Illumina, San Diego, CA, USA) according to the manufacturer’s instructions. Array scanning data were generated on the Illumina iScan system, and the results were analyzed by Bluefuse Multi 4.4 software. The confirmation and segregation tests were performed on the patient’s and her parents’ DNA by conducting real-time PCR on *CYB5R3* using an SYBR Green assay [4].

#### 2.2.2. Panel for Congenital Cerebellar Ataxias

An NGS panel of genes for which its mutations are causative of various forms of congenital cerebellar ataxias, including known PCH genes, was performed as previously described [5].

#### 2.2.3. Review of the Genetic, Neurological and Neuroimaging Literature Data on RHM2

Literature review was performed on PubMed (https://www.ncbi.nlm.nih.gov/, accessed on 15 January 2022) by using the following key terms alone or in combination: *CYB5R3*; methemoglobinemia type II; methemoglobinemia generalized; diaphorase 1; cytochrome b5 reductase 3 gene; and NADH-reductase. Articles reporting clinical data on humans were then reviewed, and reference lists were also screened for additional missed papers.

## 3. Results

### 3.1. Genetic Analysis

**Figure 3:**
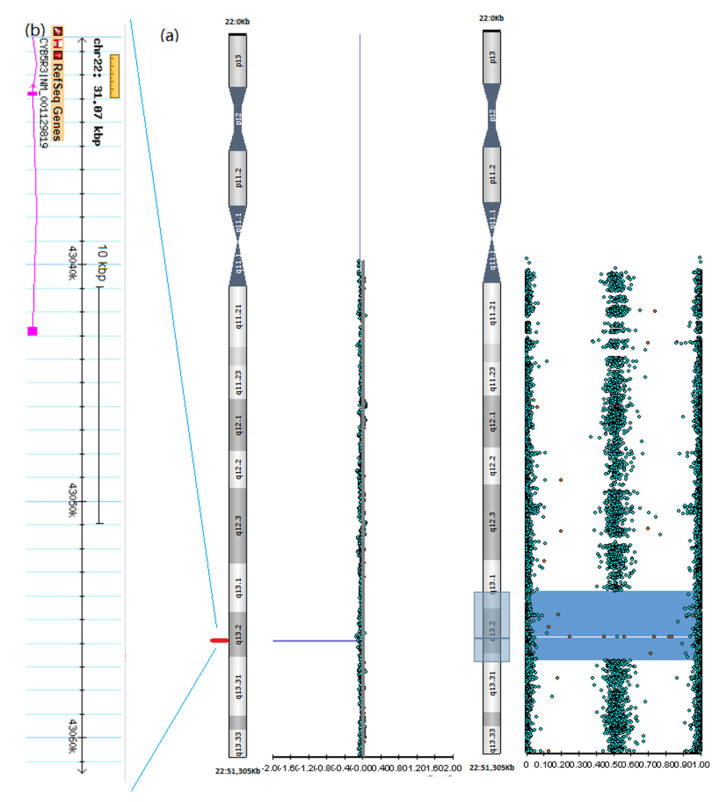
SNP-array analysis output from BlueFuse Multi software. (**a**) A 5 Mb ROH (Run of Homozygosity) is shown as a gray area on the ideogram of chromosome 22. The CNV (Copy Number Variant) and BAF (B allele frequency) graphs are reported on the left and on the right side, respectively. Homozygous deletion is represented as red non-classified spots on the BAF graph and as a red line on the CNV graph. (**b**) Enlargement of the 22q13.2 homozygous deletion, involving the first two exons of *CYB5R3*.

A homozygous microdeletion of about 31 Kb in the 22q13.2 region (chr22:43,030,430–43,061,494) was detected in the proband (Figure 3). This microdeletion, inherited from heterozygous carrier parents, involves part of *CYB5R3.* No putative pathogenic variants were detected by the NGS panel for congenital cerebellar ataxias.

### 3.2. Clinical Follow-Up

After the diagnosis of RHM2 was made, treatment with ascorbic acid at 400 mg/day was immediately started and allowed for a stable reduction in MetHb levels from 28% to 13% and the resolution of cyanosis (Figure 1b). The last neurological examination at age 22 months showed the following: mild microcephaly (head circumference of 44.3 cm, <3%); an alternating squint; improved eye contact with the ability to follow objects and to smile; improved vocalization; complete head control; upper and lower limb spasticity with increased deep osteo-tendinous reflexes; a mild truncal hypotonia with the ability to sit independently only for few seconds; and no lateral parachute reflexes. The dimension A score of the gross motor function measure 88 was of 23.52%. Weight (10.7 kg) and length (83 cm) were both within the normal range (25–50%). A video electroencephalogram showed fairly organized background activity without epileptic anomalies. The patient is currently receiving ascorbic acid at the same dosage and undergoes psychomotricity and physical therapy; no other pharmacological therapies have been administered.

### 3.3. Review of the Genetic, Neurological and Neuroimaging Literature Data on RHM2

Fifty-two articles were identified. Among these, thirty-one were available for review [1,6,7,8,9,10,11,12,13,14,15,16,17,18,19,20,21,22,23,24,25,26,27,28,29,30,31], and twenty-one were excluded (Supplementary File S1) due to a lack of original articles for direct review. Fifty-five patients were identified from the selected articles. Among these, any duplicates, i.e., the same patients described multiple times, were counted once [1,9,10,21,31,32,33,34]; one patient was erroneously diagnosed as RHM2 [16]; and data were missing in one case [34], thus generating a final number of forty-nine patients from forty-two families with RHM2 selected for final revision. Data are summarized in Table 1 and more extensively reported in Table S1.

## 4. Discussion

Although RHM2 was first described many years ago, most articles have focused on hematological and biochemical and, therefore, genetic features, while delineation of neurological aspects was mostly limited to descriptions of features such as mental retardation, microcephaly, generalized dystonia and movement disorders. This rare disorder has been observed worldwide (Supplementary File S1). Over the years, several terms, such as “cytochrome B5 reductase deficiency” [8], “recessive congenital methemoglobinemia” [10], “generalized hereditary methemoglobinemia” [12] and “methemoglobinemia reductase deficiency” [26], have been used to indicate this condition. In 2008, the name “recessive hereditary methemoglobinemia” was proposed [21], but there is still no uniformity about nomenclature. Our analysis of published articles (Table 1 and Table S1) suggests that an isolated cyanosis is the presenting sign of RHM2 in a considerable percentage (38%) of cases, although many patients may exhibit a complex phenotype with neurological involvement from onset, and sometimes cyanoses can only be recognized after the detection of neurological signs. Notably, RHM2 is the only condition to manifest neurological involvement among the inherited forms of methemoglobinemia [35]. Available data also indicate that onset is usually at birth or, in a smaller percentage of cases, within the first months of life. Similar to type I, MetHb levels are usually 20–30% [35]. Cognitive and motor delays are invariably present, while other features such as movement disorders, strabismus, microcephaly or epilepsy are not always observed. Movement disorders are of a hyperkinetic type and mostly include choreo-athetosis and dystonia or a combination of these. Epilepsy has been described in 16% of cases but, when present, it is drug-resistant in most patients. Patients may also show growth retardation exacerbated by feeding difficulties due to swallowing disturbances. Outcome data are poor, although the outcome is generally described as severe, with only a few patients reportedly able to acquire autonomous walking. Neurological involvement seems not to be progressive in the majority of cases, but life expectancy is reduced, mostly due to swallowing difficulties and respiratory complications, and death may occur in the first decade of life, although some patients survive into adulthood [1,6,7,8,9,10,11,12,13,14,15,16,17,18,19,20,21,22,23,24,25,26,27,28,29,30,31,35].

Available neuroimaging data indicate that brain atrophy is the most common finding of both computed tomography and MRI, followed by white matter anomalies, such as a reduction in the total amount of white matter and hypomyelination/delayed myelination. It is likely that hypomyelination is secondary to a primitive neuronal disorder, considering the presence of early-onset atrophy; however, descriptions of white matter anomalies result from a single brain MRI often performed in the first year of life and no images are available for review. Finally, some less frequent findings include thin corpus callosum, hypoplasia of basal ganglia or PCH-like conditions, thus potentially increasing the number of differential diagnoses. For example, tubulinopathies may show abnormalities of the corpus callosum and basal ganglia [36], while PCHs, especially some non-progressive forms such as *CASK*-related, *VLDRL*-related and *RELN*-related PCH, may show a combination of small pons and cerebellum coupled with a simplified gyral pattern [37]. From a clinical point of view, all of these forms mostly present with microcephaly, global developmental delay and spastic tetraparesis; however, a persistent cyanosis remains a unique diagnostic clue that must be considered in cases of suspected RHM2.

With regard to pathogenic variants, previous articles indicate that RHM1 is usually associated with missense variants that result in protein instability, with decreased erythrocyte CYB3R5 concentration, whereas type II is frequently caused by loss-of-function variants with enzymatically inactive proteins due to altered splicing, disruption of the enzymatic active site or premature protein truncation. So far, thirty-four different *CYB5R3* pathogenic variants causing RHM2 have been identified (Table S1). Almost all cases harbor point mutations or intragenic deletions (Table S1). Occasionally the same variant was observed in both RHM type I and II [1], thus indicating the presence of unknown modifiers. Few variants are recurrent in more than one case (e.g., c.463 + 8G>C, p.Arg58Pro, p.Gly76Ser and p.Gln77*), while most of them are private variants (Table S1). A few variants have been associated with a less severe (i.e., intermediate) phenotype [21]. Our case represents the first RHM2 caused by a copy number variation.

Therapy for both type I and II congenital methemoglobinemia consists in the administration of methylene blue when the blood MetHb level is over 30% or, in the presence of severe symptoms, with levels between 20% and 30%. Ascorbic acid or riboflavin can be used when the level of MetHb is lower or when methylene blue is unavailable or contraindicated. Methylene blue and riboflavin act as electron acceptors in the nicotinamide-adenine dinucleotide-flavin reductase system, whereas ascorbic acid is an electron donor that can directly promote a reduction in MetHb levels. In RHM2, these therapies are reported to treat cyanosis, but most articles indicate that they were quite ineffective on neurologic aspects. A few anecdotal reports [20,23], however, indicated some improvements in neurologic functions such as attention and active participation, as observed in our case. No clinical trials have been conducted [35].

## 5. Conclusions

RHM2 is a rare disease with less than sixty patients described from the mid-1950s to date. The neurological phenotype is consistent with a severe neurodevelopmental disorder characterized by global developmental delay; congenital or acquired microcephaly; tetraparesis; spasticity; extrapyramidal hyperkinetic movement disorders; and drug-resistant epilepsy in a smaller percentage of patients. The phenotype per se is not distinguishable from several neurodevelopmental syndromes with or without degenerative features. However, the presence of persistent cyanosis from birth or during the first months of age and in the absence of respiratory and cardiac involvement is a notable aspect, since it is strongly indicative of RHM2. Neuroimaging seems to be consistent with a primitive neuronal disorder (i.e., cerebral and/or cerebellar atrophy with secondary white matter anomalies), and it can give rise to difficulties in differential diagnosis when atypical patterns (i.e., PCH-like or basal ganglia hypoplasia) are encountered. The paucity of literature data on deep neurological and neuroimaging phenotyping or on outcomes after therapy indicates the need for novel systematic studies on these aspects, as well as for clinical trials to better understand the natural history and outcome of RHM2.

*CYB5R3* screening should be considered for children exhibiting complex neurodevelopmental phenotypes. If RHM2 is suspected, a search for copy number variations should be performed after sequencing *CYB5R3*.

## Figures and Tables

**Table 1:** Revision of the main features of the 50 patients with RHM2 (forty-nine patients from thirty-one available literature articles plus our case).

Features	Reported Patients
**First sign(s)/symptom(s) at onset**	
Isolated cyanosis	19/50
Cyanosis + isolated developmental delay	5/50
Cyanosis + complex phenotype	8/50
Other (without cyanosis)	10/50
Not reported	8/50
**Age at onset/recognition**	
Birth-Neonatal period	27/50
1–12 months	9/50
>1 year	-
Not reported	14/50
**Cognitive delay**	49/50
Not reported	1/50
**Motor delay**	49/50
Not reported	1/50
**Strabismus**	20/50
**Microcephaly**	33/50
Pyramidal signs *	24/50
**Extrapyramidal movement disorders**	
Dystonia	20/50
Choreo-athetosis	14/50
Non-specified hyperkinetic MD	3/50
Mixed MD	10/50
**Epilepsy**	
Responsive epilepsy	1/50
Drug-resistant epilepsy	7/50
**Neuroimaging**	
Not reported	21/50
** CT**	
Normal	2/6
Brain atrophy	4/6
** MRI**	
Normal	2/23
Brain atrophy	21/23
Cerebellar atrophy	9/23
Midbrain atrophy	1/23
Basal ganglia atrophy/hypoplasia	3/23
White matter anomalies ^	18/23
Thin corpus callosum	5/23
Gyral simplification	1/23
**Outcome**	
Severe cognitive–motor impairment °	24/50
Preserved gait	3/50
Premature death	2/50
Not reported or clearly explained	21/50
Pathogenic variants ^*	34/50

^ Include: hypomyelination/delayed myelination and white matter reduction. * Include: hypertonia, spasticity, increased deep osteo-tendineous reflexes and Babinski’s sign. ° Outcome data refer to (lack of) available information. ^* Number of different pathogenic variants; pathogenic variants have not been reported in 9 out of 49 cases.

## Data Availability

The data presented in this study are available upon reasonable request from the corresponding author.

## Supplementary Materials

The following supporting information can be downloaded at: https://www.mdpi.com/article/10.3390/brainsci12020182/s1, Table S1: Patients and articles included in the literature review. Supplementary File S1: articles excluded from literature review and geographic origin of the published patients.
