# Supplementary material for: Neurological and Neuroimaging Features of CYB5R3-Related Recessive Hereditary Methemoglobinemia Type II
Source: Brain Sci. 2022 Jan 29;12(2):182. doi: 10.3390/brainsci12020182 (PMC8870218; doi:10.3390/brainsci12020182)
Supplement: Supplementary file 1 [file brainsci-12-00182-s001.zip › Supplementary file 1.pdf]

LIST OF ARTICLES NOT INCLUDED FOR REVIEW

1. Abdemoula MS. La methemoglobinemie hereditaire recessive de type II. A propos d'une observation. Revue Maghrebine de Pediatrie 2002; XII-IV: 207–10.
2. Beauvais P, Leroux A, Kaplan JC. La methemoglobinemie hereditaire avec arrieration mentale et troubles neurologiques. Nouv Presse Med 1976 5:2793-5.
3. Fialkow BJ, Brower JA, Sparkes RS, Motuisky AG. Mental retardation in methemoglobinemia due to diaphorase deficiency. N Engl J Med 1965; 273:840-5.
4. Galeeva NM, Nenasheva SA, Kleĭmenova IS, Poliakov AV. Novel large deletion c.22-1320_633+1224del in the CYB5R3 gene from patients with hereditary methemoglobinemia. 2012;48(11):1336-46.
5. Jablonska-Skwiecinska E, Holtorp-Tyszkiewiczowa J, Staniszewska K. Generalized deficiency of the NADH-methemoglobin reductase in congenital methemoglobinemia with neurological symptoms. Biomed Biochim Acta 1984; 43: S98–100.
6. Jaffe ER. Hereditary methemoglobinemias associated with abnormalities in the metabolism of erythrocytes. Am J Med 1966; 41: 786–98.
7. Heusden A, Willems C, Lambotte C, Hainaut H, Chapelle P, Malchair R. [Hereditary methemoglobinemia with mental retardation. Study of 3 further cases]. Arch Fr Pediatr 1971; 28: 631–45.
8. Kaftory A, Freundlich E, Manaster J, Shukri A, Hegesh E. Prenatal diagnosis of congenital methemoglobinemia with mental retardation. Isr J Med Sci 1986; 22: 837–40.
9. Kaplan JC, Leroux A, Beauvais P. Clinical and biological forms of cytochrome b5 reductase deficiency. C R Seances Soc Biol Fil 1979; 173:368–79.
10. Lamy M, Frezal J, Jammet ML, Josso N. [Recessive congenital methemoglobinemia.]. Nouv Rev Fr Hematol 1963; 3: 105–20.
11. Leroux A, Junien C, Kaplan JC, Bamberg J. Generalized deficiency of cvtochrome b5 reductase in congenital methaemoglobinaemia with mental retardation. Nature 1975 ;258:619-20.
12. Lostanlen D, Vieira de Barros A, Leroux A, Kaplan JC. Soluble NADHcytochrome b5 reductase from rabbit liver cytosol: partial purification and characterization. Biochim Biophys Acta 1978; 526: 42–51
13. Nishina T, Miwa S, Hara N, Asakura T. Hereditary methemoglobinemia with mental and growth retardation found in a Japanese boy. With special reference to xylitol, sorbitol metabolism and methemoglobin reduction in the erythrocyte. Acta Haematol Jpn 1970;33:455-61.

14. Orsini A, Vovan L, Brusquet Y, Gabriel B, Sebag F, Galtier M. Congenital methemoglobinemia due to NADH (DPNH) dependent methemoglobin reductase deficiency. Mars Med 1972; 109: 279–81.
15. Ronconi G, Ferracin G. [Congenital methemoglobinemia of the recessive type due to diaphorase deficit with oligophrenia.]. Riv Clin Pediatr 1964; 74: 152–9.
16. Ronconi G, Ferracin G. On a new case of congenital methemoglobinemia caused by deficiency of diaphorease with grave cerebropathy. Fracastoro 1968; 61: 121–8.
17. Roussel A, Maestraggi P, Tremoulet M, Marchand .A new case of recessive congenital methemoglobinemia. Arch Fr Pediatr 1963; 20: 745–50.
18. Sacerdotti-Favini. Methemoglobinemia costituzionale con cerebropatia e oligofrenia. Acta pediat Lat 1948; 11: 255.
19. Shotelersuk V, Tosukhowong P, Chotivitayatarakorn P, Pongpunlert W. A Thai boy with hereditary enzymopenic methemoglobinemia type II. J Med Assoc Thai 2000; 83: 1380–6.
20. Vives-Corrons JL, Pujades A, Vela E, Corretger JM, Kaplan JC. Congenital methemoglobin reductase (cytochrome b6 reductase) deficiency associated with mental retardation in a Spanish girl. Acta Haematol 1978 ;59:348-53.
21. Williamson DA, Black JA. Congenital methaemoglobinaemia; a case report. Great Ormond St J 1954; 7: 56–61.

GEOGRAPHIC ORIGIN AMONG PATIENTS ANALYZED FROM LITERATURE REVISION

India: 13 patients

Japan: 5 patients

Turkey, France, Algeria: 3 patients each

Italy, Cuba, Saudi Arabia: 2 patients each

England, South Africa, Thailand, Suriname, Germany, Pakistan, Lebanon, Tunisia, Egypt, Honduras, Australia, USA: 1 patient each

Mixed ethnicity: French-Spanish in one patient

Not clearly reported: 4 patients
