# Supplementary material for: Neurological and Neuroimaging Features of CYB5R3-Related Recessive Hereditary Methemoglobinemia Type II
Source: Brain Sci. 2022 Jan 29;12(2):182. doi: 10.3390/brainsci12020182 (PMC8870218; doi:10.3390/brainsci12020182)
Supplement: Supplementary file 1 [file brainsci-12-00182-s001.zip › Table S1.pdf]

Table S1: Main features in patients with *CYB5R3*-related congenital methemoglobinemia type II selected from available literature data.
*In reference to symptoms and signs, original nomenclature used in the reference article has been left.
List of abbreviations: BG basal ganglia, CC corpus callosum, CI cognitive impairment, CSF cerebrospinal fluid, CT computed tomography, CP cerebral palsy, DD developmental delay, DRE drug-resistant epilepsy, MR mental retardation, Mic Microcephaly, MD movement disorders, NR not reported, OFC occipito-frontal circumference, PMD psychomotor delay, WM white matter.

**Patient (original description)**	**Ethnicity**	**Age at onset**	**Symptoms at onset***	**Other symptoms during disease's course**	**MetHB level %**	**Neuroimaging**	**EEG**	**CYB5R3 variants**<br><br>**NM_000398.7 NP_000389.1**	**Therapy and outcome (age, status, follow up MRI, MetHb)**	**Reference**
Case 4<br><br>Case 5	Cuban<br><br>Cuban	Birth<br><br>Birth	Cyanosis<br><br>Cyanosis	PMD, severe speech delay<br><br>PMD with severe speech delay	19.7<br><br>15	NR<br><br>NR	Slowly generalized<br><br>NR	NR<br><br>NR	Improvement of speech delay after 15 years<br>NR	Gonzalez et al., 1978<br><br>Gonzalez et al., 1978
III 5<br><br>III 2<br><br>III 4	Japanese	NR<br><br>NR<br><br>NR	Cyanosis, MR<br><br>Cyanosis , MR<br><br>Cyanosis, MR	NR<br><br>NR<br><br>NR	10.7<br><br>23.6<br><br>18.6	NR<br><br>NR<br><br>NR	NR<br><br>NR<br><br>NR	c.382T>C p.Ser128Pro	24 years<br><br>NR<br><br>NR	Kobaiashi et al., 1990
----	Japanese	Birth	Cyanosis (isolated)	PMD, joint malformations, short stature	15.4	Cortical atrophy (CT)	Borderline	NR	43 years, clumsy gait and mental retardation	Yawata et al., 1992
----	Japanese	Birth	Cyanosis (isolated)	PMD with poor head control (6mo), choreo-athethosis, cerebellar involvement, increased OTR	24	Normal (CT)	Normal	c.895-897del p.Phe299del	13 years no worsening of movement disorders, bizarre gait, speech disturbance, slight scoliosis	Shirabe et al., 1994<br><br>(previously described in Nishina et al., 1970 and Takeshita et al., 1982)
BEN	Algerian	NR	Profound MR Mic, bilateral athetosis	NR	NR	NR	NR	c.463+8G>C	NR	Mota Viera et al., 1995<br><br>(previously described in Kaplan et al., 1979)
BOU	Algerian	NR	Profound MR, Mic,bilateral athetosis	NR	NR	NR	NR	c.655C>T p.Arg219*	NR	Mota Viera et al., 1995<br><br>(previously described in

										Lostanlen et al., 1981)
----	Italian	Birth	Cyanosis	First months: feeding difficulties, failure to thrive, PMD<br>1 year: severe spastic-dystonic quadriparesis, hyperkinetic MD, Mic, DRE	NR	Diffuse brain atrophy (CT)	Slow and irregular basal activity with extremely frequent and periodic paroxysmal burst spike-wave complexes during sleep	c.458-1G>T	No response to Vitamin C, vegetative status at 9 years	Shirabe et al., 1995
---	United Kingdom	Early infancy	Cyanosis , DD	NR	11.3	NR	NR	c.129>A<br>p.Tyr43*<br><br>c. 287C>A<br>p.Pro96His	6 years<br>squint, mild tremor, and some degree of hypertonia	Manabe et al., 1996
---	South Africa	NR	Cyanosis, dystonic-athetoid CP, MR, Mic	NR	60	NR	NR	c.464-2A>C	NR	Owen et al., 1997
Patient 1	Thai	NR	NR	NR	NR	NR	NR	c.250C>T<br>p.Arg84*	Absent speech, no sit position, spasticity	Higasa et al., 1998
---	Hindoostani Surinam	Birth	SGA, cyanosis	Severe PMD, Mic, athetoid movements, generalized hypertonia, DRE, dysphagia	40	frontal and bitemporal cortical atrophy, cerebellar atrophy, retarded myelinization, and BG hypoplasia	NR	c.229C>T<br>p.Gln77*<br>+<br>c.478C>T<br>p.Arg160*	Died suddenly at 8 years, probably due to aspiration	Aalfs et al., 2000
Case 3	German	Birth	Cyanosis	Severe PMD	15	Slightly reduced myelination and CC atrophy	NR	c.334-2A>G<br>+<br>c.379A>G<br>p.Met127Val	4 years started walking at three years	Kugler et al., 2001
Case 1	Pakistan	Birth (normal OFC)	Cyanosis	4 months: DD, spasticity, Mic	17	4 months: enlargment of CSF spaces and delayed myelination	NR	NR	4 years: Mic, short stature, strabismus, failure to thrive, profound CI, no language, spastic tetraparesis (unable to sit)	Toelle et al., 2004
Case 2	Turkish	Birth (normal OFC)	Cyanosis	4 months: DD	35	5 and 16 months: enlargment of CSF spaces and delayed myelination, thin CC	NR	c.721A>G<br>p.Arg241Gly	2.5 spastic tetraparesis (unable to sit), profound CI, strabismus, Mic	Toelle et al., 2004
----	NR	Birth	Cyanosis	DD, Mic	16.3	NR	NR	c.463+2T>C	5 years<br>No head control	Yilmaz et al., 2005

----	NR	3 months	Cyanosis and agitation	1.5 years: DD, hypotonia, no head control<br><br>4 years: atonic convulsion episodes	51	10 years MRI normal	Disorganization	NR	10 years Mic, strabismus, severe CI, tetraparesis, no language, can sit with help, dysphagia (improvement of dysphagia and social contact after ascorbic acid)	Gokalp et al., 2005
Case 1<br><br>(patient LAM in Mota Viera et al., 1995)	French and Spanish	14 days	Cyanosis, vomiting, Mic, Dystonia, distal upper limb pronation, motor uncordination	PMD, dystonia, athetosis, strabismus	15	(CT) cortico-subcortical atrophy with prominent involvement of the frontal and temporal lobes	diffuse slowing with no epileptic features	c.610T>C p.Cys204Arg + c. 815-817del p.Met273del	Early death (age not specified)	Ewenckyz et al., 2008<br><br>(previously described in in Mota Viera et al., 1995)
Case 2	French	2 days	Cyanosis	Global hypertonia with flexion spasms, athetosis (10mo). Mic (11mo), strabismus	32	NR	normal	c.392T>C p.Leu131Pro + c.226G>A p.Gly76Ser	Living in a specialized institute for severely disabled persons. Speech a gross motor function delay.	Ewenckyz et al., 2008
Case 3	Lebanese	4 month	Mic (at birth - 2DS) axial hypotonia with limb hypertonia	Hypotrophic, cyanosis, dystonia, athetosis, strabismus pyramidal signs, gross motor delay	28,4	cortical atrophy	normal	c.882-884delinsAA p.Thr295fs*	Death at age 10,5 years of pneumonia.	Ewenckyz et al., 2008<br><br>(previously described in Leroux et al., 2005)
Case 4	Tunisian	Birth	Mic	Global hypotonia, profound CI, strabismus, generalized dystonia, athetosis.	17,5	cerebral atrophy. Normal spectroscopy	diffuse slowing with no epileptic features.	c.463+8G>C	10 years hypotrophic, with MD, improvement in reactivity to environmental stimuli. Speech impairment	Ewenckyz et al., 2008
Case 5	French	1 month	Generalized hypotonia, dystonia, failure to thrive, strabismusm vomiting	Mic, axial hypotonia, general dystonia, diffuse choreo-athetosis (2y). Speech delay. West syndrome, strabismus	23,7	cranial dysmorphy, global cerebral atrophy and delayed myelination. Spectro-MRI was normal.	hypsarrhythmia	c.332A>T p.Lys111Met	At age 3.5 years, mic had worsened. Improvement in choreo-athetoid movements, but increased in dystonic postures.	Ewenckyz et al., 2008

Case 6	French	Birth	Cyanosis	Mic (5 months), axial hypotonia, peripheral hypertonia with athetoid arm movements, strabismus (6 months). He could not sit, but made sounds, smiled and reacted to his name	23	NR	NR	c.153+1G>A	18 months. Progressive mic, PMD with partial improvement in swallowing ability	Ewenckyz et al., 2008
Case 1	Egyptian	5 months	Cyanosis, DD, profound CI, mic, bilateral athetosis, strabismus, Vomiting, crying	NR	12,1	NR	NR	c.153+2T>C	5 m: ascorbic acid therapy	Fermo et al., 2008
---	Honduran	Birth	Cyanosis	1 months: hypertonia than hypotonia. Infancy: Severe growth retardation and global neurologic impairments strabismus, limbs dystonia and choreo-athetosis	21,3	diffuse atrophy and decreased WM	NR	c.215delG p.Gly72Alafs*100.	Last FU 8 years. 500 mg of oral ascorbic acid per day. After 9m mild-to-moderate improvements in multiple parameters, especially in muscle strength and coordination related to sitting, chewing, and swallowing	Hudspeth et al., 2009
Case 1	Turkish	Birth	Cyanosis	2y: severe DD (inability to lag head, sit, and crawl, grasp objects or talk), mic, frequent vomiting	28	cerebral atrophy, retarded myelinization, and thin CC	NR	NR	2y: ascorbic acid (500 mg daily) was started.	Yuksel et al., 2009
Case 2		Birth	Cyanosis	1y: severe DD, growth retardation. mic, strabismus, generalized hypertonia.	26	cerebral atrophy, retarded myelinization, thin CC	NR	NR	1y: ascorbic acid (500 mg daily) was started.	
----	Algerian	Birth	Hypotonia, mic, small for gestational age	6m: severe hypotonia, strabismus, profound cognitive impairment, areflexia, generalized dystonia with choreoathetoid arm	22	At 1 year normal. At 4 year diffuse cortical-subcortical atrophy with prominent involvement of the frontal lobes as well as delay of myelination in the	6 month: diffuse slowing of the background activity with no epileptic features	c.463+8G>C	5 years<br><br>Ascorbic acid (100 mg/kg/day) was started with no improvement in motor or mental performance, but resolution in the epileptic-	Fusco et al., 2011

				movements.<br><br>5 years of age: severe hypotonia (he could not control his head or sit up), progressive severe mic (-7 SD), strabismus, global dystonia with severe limb choreo-athetosis, facial grimacing, growth retardation, cyanosis, paroxysmal episodes resembling epileptic spasms.		hemispheric white matter. Global cerebellar atrophy.			type spasms.	
Case II-4		Birth	Cyanosis	NR	51.4	NR	NR		6 years:<br>DD, severe tetraspastic CP, profound CI, secondary mic, generalized dystonia	
Case II-1	Indian	Birth	Cyanosis	NR	12.68	NR	NR	c.611G>A<br>p.Cys204Tyr<br>+<br>c.708G>A<br>p.Trp236*	13 years:<br>DD, severe tetraspastic CP, profound CI, secondary mic, generalized dystonia	Kedar et al., 2011
Case II-3		Birth	Cyanosis	NR	41.76	NR	NR		8 years: DD, severe tetraspastic CP, profound CI, secondary mic, generalized dystonia	
Case 1	Australian	Birth	Periodic dusky episodes and skin mottling	12 weeks: poor feeding<br>4 months: failure to thrive, mild central hypotonia with head lag, axial hypotonia.<br><br>At 12 weeks ascorbic acid 250mg daily was started	17,5	16 months: normal for age	NR	c.173G>C<br>p.Arg58Pro<br>+<br>c.226G>A<br>p.Gly76Ser	Last FU: 20 months of age. He can sit independently and have a pincer grip.<br>He babbles, but has no words. Thoracic scoliosis with dystonia	Percy et al., 2012
Case 2	NR	Birth	Cyanosis	NR	10,4-30	NR	NR	c.173G>C<br>p.Arg58Pro<br>+	2 years 5 months: PMD	Percy et al., 2012

								c.562_564del p.Leu188del		
Case 19	Indian	NR	NR	severe DD, mic, strabismus, spasticity, dystonia	12	generalized brain atrophy, atrophy of the cerebellum, mild hypomyelination.	NR	c.226G>A p.Gly76Ser	3 y methylene blue (0.5 mg/kg body weight)	Warang et al., 2015a
Case 20	Indian	NR	NR	severe DD, mic, strabismus, spasticity, dystonia	72	generalized brain atrophy, atrophy of the cerebellum, mild hypomyelination	NR	c.226G>A p.Gly76Ser	2 years and a half. methylene blue (0.5 mg/kg body weight)	Warang et al., 2015a
Case 21	Indian	NR	NR	severe DD, mic, strabismus, spasticity, dystonia	15,2	generalized brain atrophy, atrophy of the cerebellum, mild hypomyelination	NR	c.708G>A p.Trp236*	3 years and a half. methylene blue (0.5 mg/kg body weight)	Warang et al., 2015a
Case 22	Indian	NR	NR	severe DD, mic, strabismus, spasticity, dystonia	52	generalized brain atrophy, atrophy of the cerebellum, mild hypomyelination	NR	NR	3 years. methylene blue (0.5 mg/kg body weight)	Warang et al., 2015a
Case 23	Indian	NR	NR	severe DD, mic, strabismus, spasticity, dystonia	25,5	generalized brain atrophy, atrophy of the cerebellum, mild hypomyelination	NR	NR	5 years methylene blue (0.5 mg/kg body weight)	Warang et al., 2015a
---	Indian	6 m	Cyanosis, DD, failure to thrive, hypotonia, mic	NR	61,1	NR	NR	c.517_525del<br><br>p.Lys173_Val175del	11 months<br><br>Treated with methylene blue (0.5 mg/kg body weight) was started with no improvement in mental performance.	Warang et al., 2015b
---	NR	NR	NR	3 year 9 months: growth and speech delay, hypotonic but could pull to stand.	14	NR	NR	c.173G>C p.Arg58Pro + c.226G>A p.Gly76Ser.	Methylene blue 1 mg/kg via gastrostomy, three times per week for 4 months, with mild improvement in behaviors problems	Cooper et al., 2016
---	American	8 days	Feeding Difficulty, frequent vomiting.	2 months: poor weight gain, lack of a social smile, hypotonia<br>3 months: mic, failure to thrive, swallowing disturbances	NR	Progressive cerebral and cerebellar atrophy, hypomyelination<br><br>(MRI performed at 5 months, 13	17 months: disorganization, multifocal and generalized spikes and spike-wave complexes	c.478C>T p.Arg160* + heterozygous partial deletion of intron 1	28 months: ketogenic diet with partial seizure control<br><br>3years 5months: decreased muscle mass, appendicular	Mannino et al., 2017

				7 months: hyperkinetic MD, spasticity, dystonia<br>12 months: dysmotility and visceral hyperalgesia<br>17 months: spastic movements, loss of head control<br>34 months: cyanosis		months, 3 year and half			hypertonia, axial hypotonia, myoclonic jerks	
Index case 1	Indian	Birth-first days	Mic, low weight. cyanosis, vomiting	First months: feeding difficulties, failure to thrive, PMD<br>1 year: spastic-dystonic quadriparesis, hyperkinetic MD, DRE	65	1 year:<br>Diffuse brain atrophy (CT)	1 year: slow and irregular basal activity with extremely frequent and periodic paroxysmal burst spike-wave complexes during sleep	c.606_607del<br>p.Thr 202Serfs*98	2 years: severe cognitive and motor impairment	Gupta et al., 2018<br><br>and<br><br>Gupta et al., 2020
Index case 2	Muslim	Birth	Mic, low weight, cyanosis	Infancy: severe growth retardation and PMD	34	Diffuse atrophy and decreased WM	NR	c.766_768del<br>p.Glu256del	10 years	Gupta et al., 2018<br><br>Gupta et al., 2020
Index case 3	Indian-Muslim	4 months	Afebrile convulsion	6 months: DRE, PMD, poor growth with OFC 97%,	39,4	Normal (CT)	Abnormal left temporal spikes	c.226G>A<br>p.Gly76Ser	3 years	Gupta et al., 2018<br>and<br>Gupta et al., 2020
Case 1	Arabian	3 months	Hypertonia in the limbs	6 months: DRE<br>22m: axial hypotonia with severe head lag, mildly increased appendicular tone with exaggerated OTR<br><br>Coenzyme Q10 and ascorbic acid therapy was started at 22months of age	16,6	10m: mild fronto-temporal atrophy. Hypoplastic BG (caudate and lentiform nuclei)<br><br>MR spectroscopy normal	NR	c.274C>T<br>p.Arg92Trp	3 years:<br>DRE, severe DD, spastic quadriplegia, axial hypotonia, hyperreflexia, strabismus. Severe oropharyngeal dysphagia with recurrent respiratory infections.	Nicolas-Jilwan, 2019

Case 2		Birth	Cyanosis	8w: severe axial hypotonia and appendicular hypertonia (dystonia and spasticity), mic, short stature<br><br>8w: ascorbic acid and coenzyme Q-10<br><br>7 months: partially-matched unrelated cord blood transplant which failed.	18	8w: mild frontotemporal atrophy, hypoplastic BG(caudate and lentiform nuclei)<br><br>Normal MR spectroscopy	NR		19 months: profound DD, spasticity, mic.<br>G-tube feeding due to severe oropharyngeal dysphagia.	
19	Indian	NR	NR	NR	12,89	NR	NR	c.229C>T p.Gln77*	10 months	Gupta et al., 2020
----	Italian	7 months	Cyanosis and PMD	1 year: strabismus, tetraparesis	20-28	PCH-like appearence, thin CC, simplified gyral pattern with reduction of frontal gyri and enlargement of sub-arachnoids spaces, WM reduction	Fairly organized bakground activity with no epileptic anomalies	Homozygous microdeletion of about 31 Kb involving part of *CYB5R3*	22 months: strabismus, spastic tetraparesis, microcephaly, improvement of eye contact and vocalization after acid ascorbic therapy.	This paper
